# Detect the early-warning signals of diseases based on signaling pathway perturbations on a single sample

**DOI:** 10.1186/s12859-021-04286-2

**Published:** 2022-01-20

**Authors:** Yanhao Huo, Geng Zhao, Luoshan Ruan, Peng Xu, Gang Fang, Fengyue Zhang, Zhenshen Bao, Xin Li

**Affiliations:** 1 Institute of Computational Science and Technology, Guangzhou University, Guangzhou, 510006 Guangdong China; 2 Netease Youdao Information Technology (Hangzhou) Co., Ltd., Hangzhou, 310000 Zhejiang China; 3 School of Computer Science of Information Technology, Qiannan Normal University for Nationalities, Duyun, 558000 Guizhou China; 4 Department of Biomedical Engineering, School of Life Science and Technology, Beijing Institute of Technology, Beijing, 100081 China; 5 Department of Gynecology, Renmin Hospital of Wuhan University, Wuhan, 430000 Hubei China

**Keywords:** Pre-disease state, Early warning signals, Single sample, Signal perturbation, Signaling pathway

## Abstract

**Background:**

During the pathogenesisof complex diseases, a sudden health deterioration will occur as results of the cumulative effect of various internal or external factors. The prediction of an early warning signal (pre-disease state) before such deterioration is very important in clinical practice, especially for a single sample. The single-sample landscape entropy (SLE) was proposed to tackle this issue. However, the PPI used in SLE was lack of definite biological meanings. Besides, the calculation of multiple correlations based on limited reference samples in SLE is time-consuming and suspect.

**Results:**

Abnormal signals generally exert their effect through the static definite biological functions in signaling pathways across the development of diseases. Thus, it is a natural way to study the propagation of the early-warning signals based on the signaling pathways in the KEGG database. In this paper, we propose a signaling perturbation method named SSP, to study the early-warning signal in signaling pathways for single dynamic time-series data. Results in three real datasets including the influenza virus infection, lung adenocarcinoma, and acute lung injury show that the proposed SSP outperformed the SLE. Moreover, the early-warning signal can be detected by one important signaling pathway PI3K-Akt.

**Conclusions:**

These results all indicate that the static model in pathways could simplify the detection of the early-warning signals.

## Background

Theoretical considerations and computational studies suggest that many types of complex dynamical systems may have a critical points between an ordered and a disordered dynamical regime [1, 2]. This regime provides complex systems to have an optimal balance between robustness and adaptability. Specifically, they can execute normal functions in a variable environment while being responsive to specific changes in the environment. Studies of human brain oscillations [3], computer network traffic and the Internet [4, 5], financial markets [6], forest fires [7], ecosystems [8, 9], climate systems [10, 11], economics and global finance [12], neuronal networks [13], and biological macroevolution have all revealed such critical dynamics [14].

Mathematically, the dynamic behavior of complex systems can be characterized by state space. One state can be represented as the behavior of the composite elements in the system. In a relatively long time, some initial states of the system will eventually settle down to one of a limited set of stable states. Bifurcation theory demonstrates that complex systems may undergo a sudden state transition under some critical continuous perturbations of various internal or externals. Such a change often occurs at a critical threshold, or the so-called ‘‘tipping point’’, at which the system shifts abruptly from one state to another.

The time evolution of complex diseases may follow the bifurcation theory, that is, a sudden health deterioration will occur during these diseases’ gradual progression at a tripping point time [15–17]. For example, it takes at least a decade or even decades for cancers to develop before getting worse [18, 19]. According to these concepts, the disease progression can be divided into three states: the normal state, the disease state, and the pre-disease state between them [20]. The normal state represents a relatively healthy stage during which the disease is under control, in an incubation period or a chronic inflammation period. The disease stage represents a continuous health deterioration that is hard to reverse. The pre-disease stage is a relatively unstable and reversible state which may transition to the normal state if appropriate treatment is applied [17, 21–25]. From the perspective of disease prevention and a better understanding of disease progression, detecting the early-warning signals for the pre-disease state is an important issue in clinical practice [17].

The execution of various physiological processes in cells is carried out by complex biomolecular systems. The massively parallel dynamics of complex molecular networks furnish the cell with the ability to process information from its environment and mount appropriate responses [26, 27]. Therefore, one direct way is determining the pre-disease state based on the state transition based on a complex network model. However, this is limited by the curse of a high dimension of genomic data and the lack of data for a single sample. The model-free direction is to identify a group of biomarkers and define a measurement based on the single sample expression data. Due to the rapid development of high-throughput technologies, innovative biomarkers are identified as the unprecedented rich information of genotypes and phenotypes of diseases. Molecular biomarkers, for example, genes, RNAs, proteins, and metabolites, are widely adopted traditional model-driven method and still play an important role in analyzing data, because of their simplicity of measurement and implementation [28–33]. Because the progression process of diseases is affected by the complications and variations of genetic, epigenetic, and environmental factors, the molecular biomarkers have a high false-positive rate and low coverage. To surmount these shortages, network biomarkers are proposed [34, 35]. Network biomarkers are considered to be more robust because a complex disease is generally caused not by the malfunction of individual molecules but by a network that contains the interplay of a group of correlated molecules [36]. However, molecular biomarkers and network biomarkers are mainly used to distinguish the disease state from the normal state by the great distinction between them [22]. Thus, they are not suitable for detecting the early-warning signals. To solve this problem, Chen and Liu et al. proposed a dynamic network biomarker (DNB), which could be applied to different application scenarios. The DNBs-based method and its subsequent modifications have been used to identify the pre-disease states of several diseases [37–41]. These methods have also been applied to detect the tipping points of cell fate decision and differentiation [42, 43], and the immune checkpoint blockade [44]. A DNB must appear and satisfy three statistic conditions which require multiple samples from an individual [17]: i.e. correlations between the variables among this group rapidly increase, correlations between this group and other variables rapidly decrease and standard deviations of the variables among this group drastically increase. The calculation of correlations and standard deviations limits the application of DNBs-based methods not fit to single case samples in clinical practice but appropriate for multiple samples. Recently, Liu et.al. proposed a new conception named the single-sample landscape entropy (SLE) based on DNB theory to detect the early-warning signals of diseases (detailed information can be seen section materials and method) [15]. The limitation of SLE lies in the following two aspects. First, the lack of definite biological meanings in PPI makes SLE indirectly depict the deviation of an individual at a time point from the health state based on the Pearson correlation change. Second, the calculation of various correlations between genes using reference samples from an individual is time consuming and suspect.

Signaling pathways in the KEGG database include a series of validated enzymatic reactions from biological experiments. And they can transmit the extracellular molecular signals into cells to exert effects through specific biological functions. The development of diseases results from the continuous perturbation of the various abnormal signals in some of these pathways. Therefore, Tarca et al. proposed a signaling pathway impact analysis (SPIA) method to determine significant disease-related signaling pathways by normal and disease samples [45]. From the perspective of system biology, signaling pathways are a kind of static biological model of the corresponding functions. In this paper, we adapt the signal perturbation to study the propagation of the early-warning signals based on the signaling pathways in the KEGG database. We name it as single-sample signal perturbation (SSP). We applied SSP to three datasets (an individual-sample dataset of influenza virus infection, the TCGA dataset of lung adenocarcinoma, and a dataset of acute lung injury). our results show that SSP outperforms SLE in predicting the early-warning signals. Also, SSP is relatively simple to avoid calculating the correlation between genes in the large PPI network. Furthermore, SSP can be further simplified by using some crucial pathways, i.e., the PI3K-Akt signaling pathway.

## Results

Complex diseases arise is due to the accumulation of differential expressions and signal perturbations in a subgroup of genes allowing uncontrolled biological functions. The accumulation of differential expressions and signal perturbations in signaling pathways is an invertible dynamic process before the biological functions become uncontrolled. Thus, when the accumulation changes in signaling pathways, it will signal a piece of pre-disease information. We use three single-sample datasets to illustrate how SSP works, including influenza virus infection (GSE30550), lung adenocarcinoma (LUAD) from the TCGA database, and acute lung injury (GSE2565). In this paper, we download 178 signaling pathways of human and 174 mmu signaling pathways from the KEGG PATHWAY dataset (https://www.kegg.jp/kegg/pathway.html). The pathways in KEGG dataset are all hand-painted and validated by scientific pieces of literature. In this section, we compare the early-warning signals detection of the proposed SSP with SLE using all these signaling pathways.

The PI3K-Akt is an important signaling pathway associated with many complex diseases, such as the later stages of influenza virus infection [46], acute lung injury [47–49], non-small cell lung cancer [50]. To demonstrate the simplicity of the proposed SSP method, we also present the predicted results on the three datasets by only using this pathway instead of all the signaling pathways.

### Early-warning signals of individual influenza infection

**Fig. 1:**
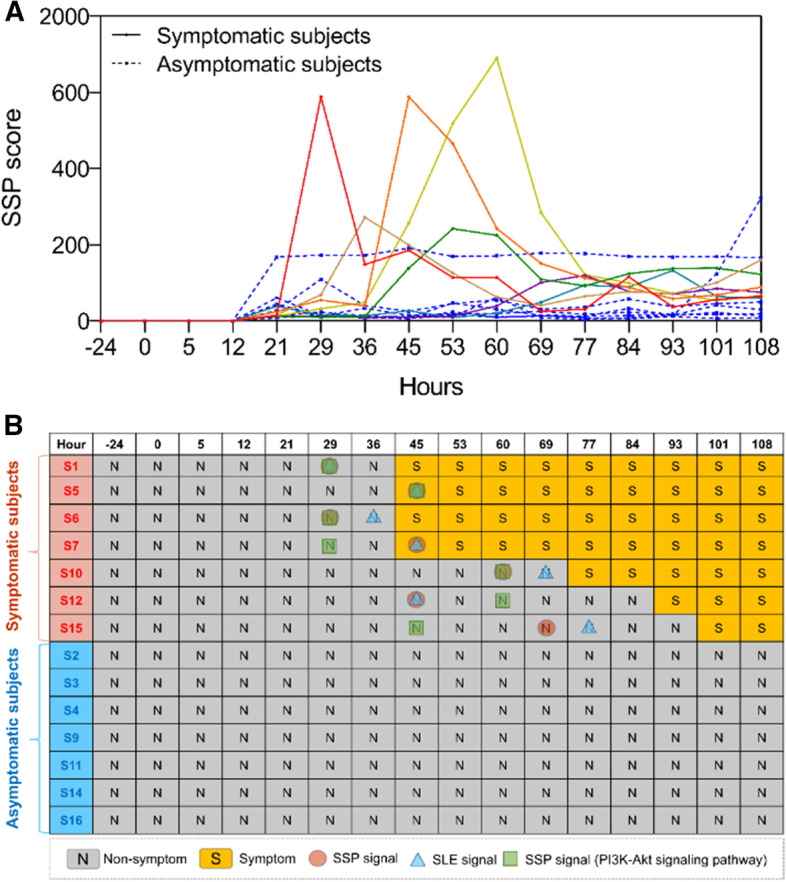
Detecting the early-warning signals of H1N2 influenza infection. **A** The curves of SSP scores for 14 subjects using 178 human signaling pathways. Each blue curve corresponds to an asymptomatic individual, while other curves record for the SSP scores based on the individual data of symptomatic subjects. **B** The summarized prediction results

For each subject in the individual influenza infection dataset (GSE30550), the gene expression profiles of the first four time points, i.e., Baseline, 0, 5, and 12 h are regarded as reference samples. Figure 1A shows the global SSP scores of the 14 subjects by all signaling pathways. First, the SSP scores of the symptomatic subjects are relatively higher and more unstable than that of the asymptomatic subjects. Second, there is a drastic increase of SSP scores of the symptomatic subjects at some middle time points, which provides an early-warning signal for these subjects. Figure 1A shows the predicted warning signals for the 14 subjects of SLE, SSP with all signaling pathways, and SSP with only PI3K-Akt pathway.

Figure 1B presents the predicted warning signals by the SLE method, SSP with all signaling pathways, and only the PI3K-Akt pathway. For the seven symptomatic subjects, three predicted warning signals by SSP with all signaling pathways are earlier than that by the SLE method. The other four warning signals are predicted at the same time point which including two signals are overlapped with the appearance of the symptom. For the SSP with only PI3K-Akt pathway, four predicted warning signals are earlier than that by the SLE method, one later and two equals. These observations indicate that the proposed SSP method has a better performance than the SLE method.

**Fig. 2:**
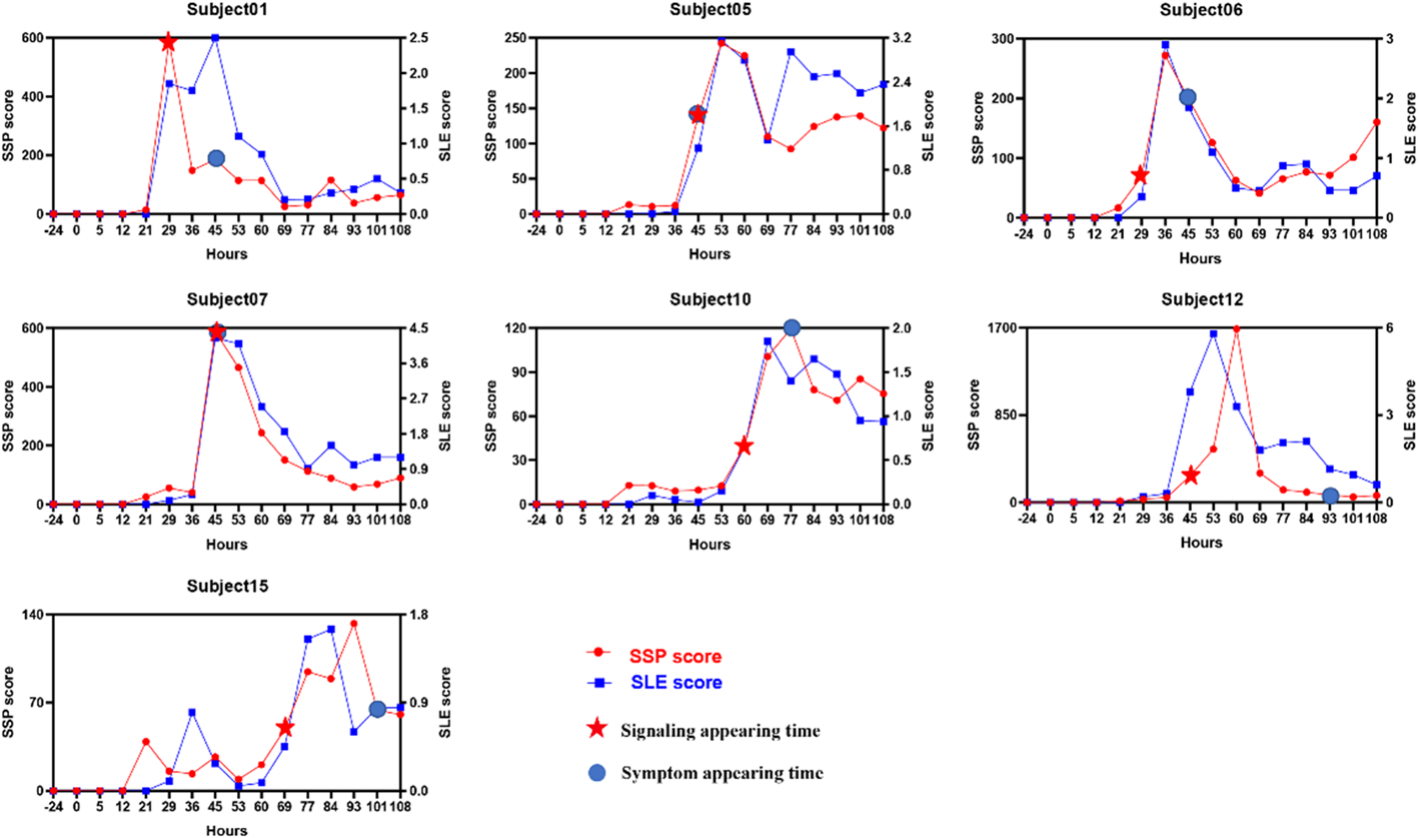
The individual SSP score curves of 7 symptomatic subjects using 178 human signaling pathways. For each symptomatic subject, the blue circle stands for the time point at which the initial flu symptoms arise, and the red star mark denotes the identified tipping point by SSP score

**Fig. 3:**
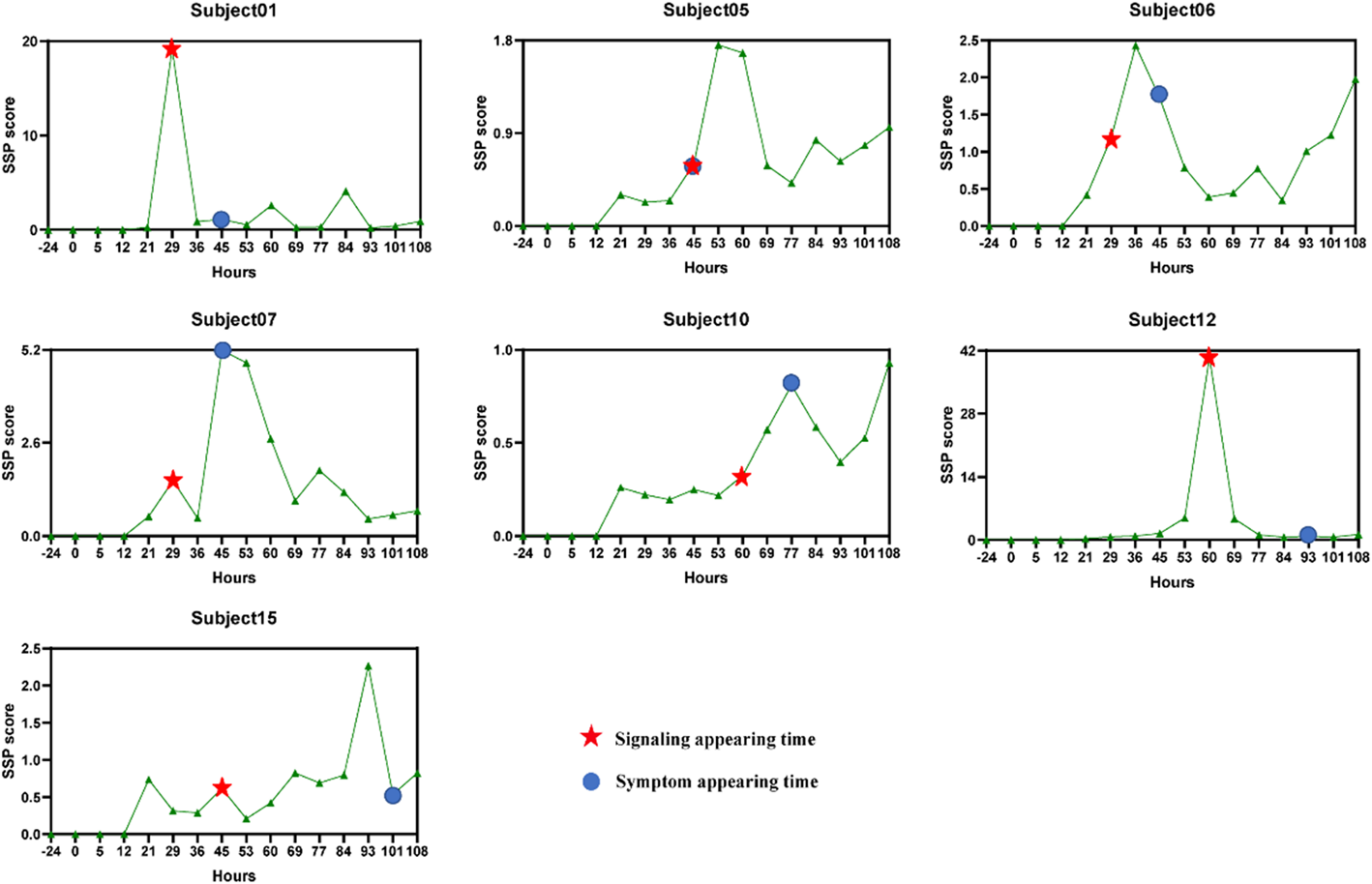
The individual SSP score curves of 7 symptomatic subjects according to the PI3K-Akt signaling pathway. For each symptomatic subject, the red circle stands for the time point at which the initial flu symptoms arise, and the blue star mark denotes the detection of the early-warning signal by SSP score

Figure 2 shows the curves of the SSP scores and SLE scores of the seven symptomatic subjects with times. The two score curves present a similar trend across all time points. Figure 3 shows the curves of the SSP scores by only PI3K-Akt pathway with times which also has a similar trend with the previous two. Compared with the SLE method, the proposed SSP methods capture the significant change of the development of diseases based on the signal perturbation in signaling pathways while the former detect it based on the correlation change between genes. Despite their difference, the results indicate that the two kinds of scores could detect the underlying molecular interaction changes in the development of diseases. However, the SSP score is easier to understand as it directly integrates the underlying molecular interactions models. Therefore, this score is more believable and easier to understand than the SLE scores.

**Fig. 4:**
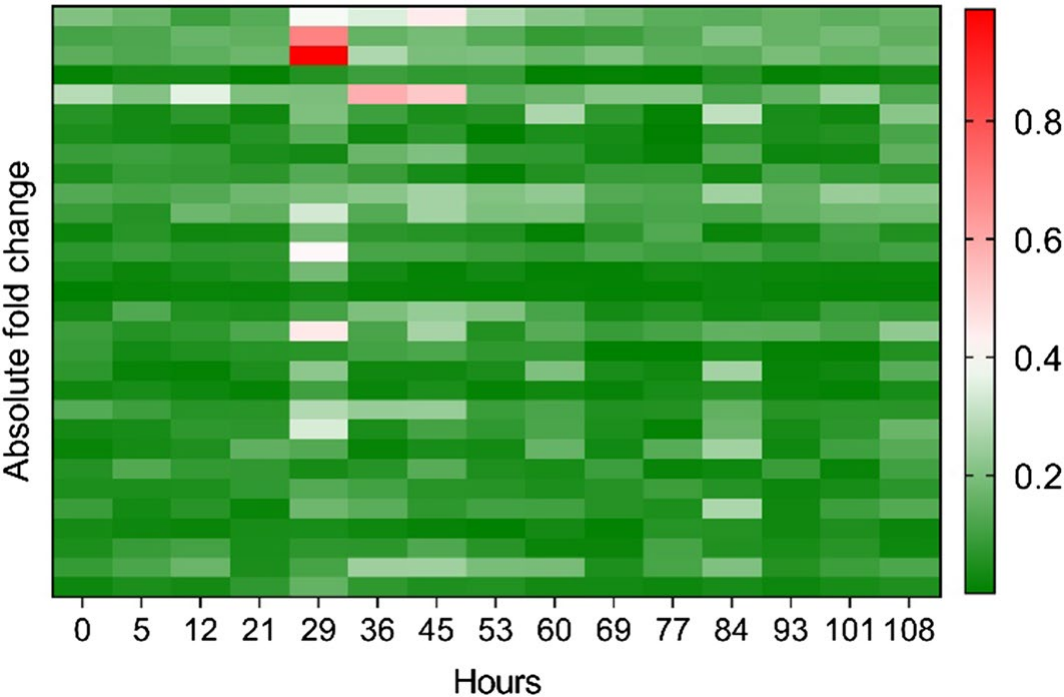
the absolute fold change value of the expression data of the genes in the upstream of PI3K-Akt signaling pathway using the individual sample Subject01

As the perturbations of upstream genes usually contribute abnormal signals in a signaling pathway, we present the fold change of the 30 upstream genes in the PI3K-Akt signaling pathway in Fig. 4 by Subject 01. We can see that some genes are significantly highly expressed at time 29 h, which is consistent with the detected warning signal for Subject01 in Fig. 3. Therefore, the pathway-based method may further help to uncover the underlying mechanisms for the pre-disease state and to predict a more precise warning signal in the future.

### Early-warning signals of lung adenocarcinoma (LUAD)

For dataset LUAD, the 58 tumor adjacent (TA) samples are considered as reference samples. Because of no individual-based samples across all time points, the expressions of genes are obtained by their average value in each time point. Metastasis is the culprit behind most cancer-related deaths and the ultimate challenge in our effort to fight cancer as a life-threatening disease [51]. Stage II means cancer may have spread from the lung to the nearby lymph nodes and stage IV means the tumor cells have invaded into distant tissues of other organs [52].

**Fig. 5:**
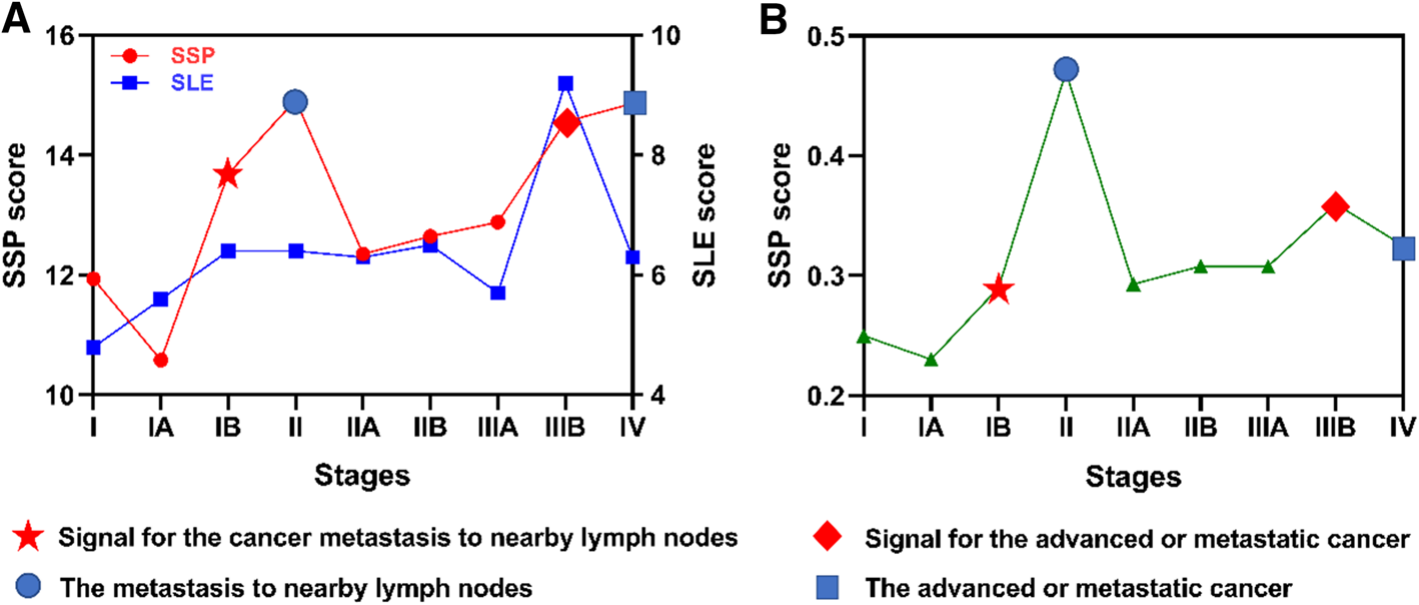
Detecting the early-warning signal of lung adenocarcinoma. **A** SSP score and SLE score curves of LUAD progression for 178 signaling pathways, which shows the early-warning signal around IA–IB stages and IIIA-IIIB stages. **B** SSP score curve of LUAD progression for PI3K-Akt signaling pathway, which shows the early-warning signal around IA–IB stages and IIIA-IIIB stages

Figure 5A shows the predicted warning signals by the SLE and SPP with all signaling pathways. the former only correctly give the warning signals before stage IV, while the latter can correctly give the warning signals for both stage II and IV. Figure 5B shows that the same results can even be obtained by SSP with only PI3K-Akt pathway. Therefore, the proposed SSP method gives better prediction performance than the SLE method. In practice, the precise warning signals will help to take chemotherapy and radiotherapy timely to prevent serious deterioration or slow down cancer progression [53].

### Early-warning signals of acute lung injury

For the acute lung injury dataset GSE2565, the samples collected from the air- or phosgene-exposed mice at 0 h are considered as reference samples. Because of no individual-based samples across all time points, the expressions of genes are obtained by their average value in each time point.

**Fig. 6:**
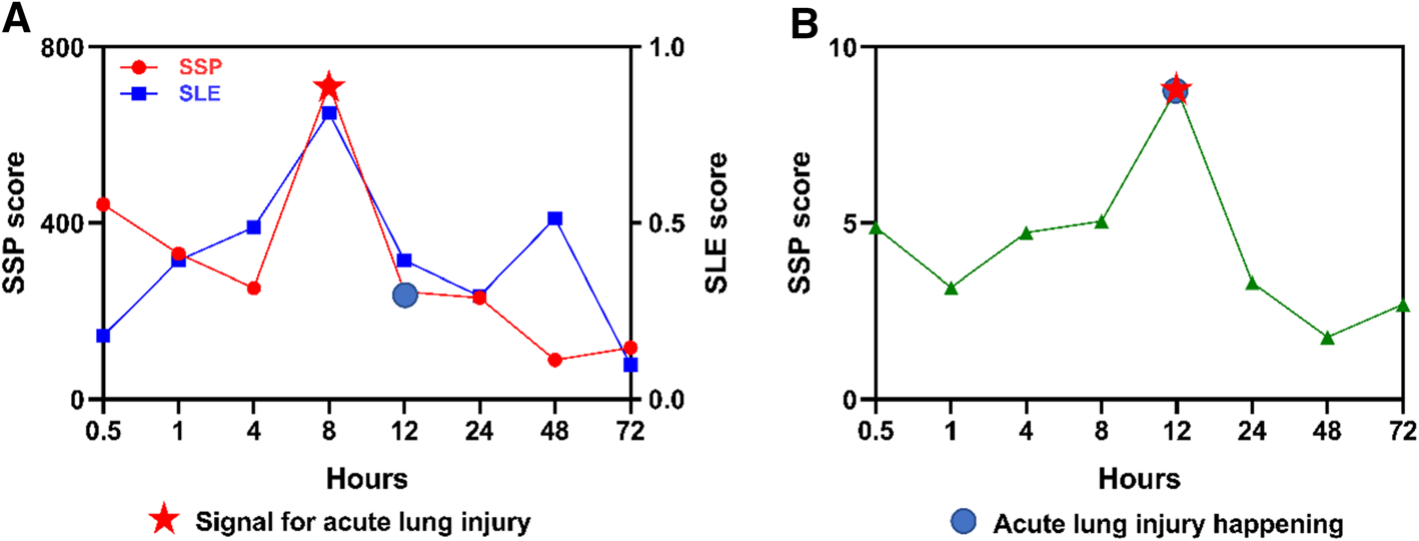
Detecting the early-warning signal of acute lung injury. **A** SSP score and SLE score curves of acute lung injury progression for 174 mmu signaling pathways, which shows the early-warning signal is around 4 to 8 h. **B** SSP score curve of acute lung injury progression for PI3K-Akt signaling pathway, which shows the early-warning signal is around 8 to 12 h

Figure 6A shows that both the SLE and SPP with all signaling pathways predict the warning signals at 8 h after exposure which is 4 h before the happening of acute lung injury. Figure 6B shows the predicted warning signal just at the observed happening time by SSP with only the PI3K-Akt pathway. The original experiment found that the most severe phosgene-induced acute lung injury ranged from 4 to 12 h after exposure. Especially, the main physiological effects occurred within the first 8 h after exposure, resulting in common observations of enhanced bronchi alveolar lavage fluid (BALF) protein levels, increased pulmonary edema, and ultimately decreased survival rates [54]. Therefore, the predicted signals are consistent with the actual disease development. In addition, in Fig. 6A, b, the SSP score from time point 0.5 to 8 h has a down behavior. The expressions of genes are obtained by their average value of all samples at the time point in each time point. Thus, the noise may be introduced in the SSP score and will result in this case. But this case does not influence the predicted signals.

## Discussion

In the progression of complex diseases, a sudden health deterioration (pre-disease state) usually occurs due to the gradual accumulating effect of various internal or external factors. An early warning signal before this deterioration is essentially important for clinical practice. Some effective treatments could be applied to prevent the happening of the irreversible process of diseases. In recent years, Chen et al. developed a new framework to tackle this issue. Especially the SLE method can detect early warning signals based on the time point expression data of an individual through the correlation changes between genes.

In this paper, we apply the signal perturbation in pathways to this framework to predict the early warning signals of complex diseases. Results from three datasets demonstrate that the proposed method SSP outperforms the SLE method in both the influenza virus infection dataset and LUAD dataset, and equals in the acute lung injury dataset. The consideration of the static model of pathways provides the proposed SSP method a relatively concrete biological mechanism to detect the essential changes. In addition, it reduces the computation complexity of the proposed SSP compared with the SLE method. Finally, based on the result by only the PI3K-Akt signaling pathway, the proposed method SSP can be further simplified by using some crucial pathways if enough data is available.

However, the SSP method still has its limitations. SSP mainly relies on the signaling pathways from KEGG datasets. The biological information included in these pathways is incomplete. Thus, the SSP score may have a fluctuation under some circumstances. The noise in initial gene expression data is also the reason for the fluctuation of the SSP score. In addition, the strength of the interactions between genes in pathways is not considered in SSP. This may result in the loss of some real biological information.

## Conclusions

In this paper, a method called SSP to identify the pre-disease state of complex diseases is presented. SSP adapted a static biological model (signaling pathways with specific biological functions) to a dynamic system to character the dynamic change of system for an individual sample, which has a better performace than previous method SLE. Besides, benefiting from the use of static signaling pathways, the calculation of SSP method takes less time than previous method SLE. Therefore, SSP can identify the pre-disease state in less time that previous methods cannot.

## Methods

### Datasets

We download three time-course or stage-course datasets including the the microarray data of influenza virus infection process (GSE30550) and acute lung injury induced by phosgene inhalation(GSE2565) from the NCBI GEO database (www.ncbi.nlm.nih.gov/geo), and data of the lung adenocarcinoma (LUAD) from the TCGA database. For all these omics genomic data, we discard the probes without the corresponding NCBI Entrez gene symbol. For each gene mapped by multiple probes, the average value is employed as the gene expression.

The dataset GSE30550 (https://www.ncbi.nlm.nih.gov/geo/query/acc.cgi?acc=GSE30550) records influenza virus infection of 16 human adult subjects inoculated with live H3N2/ Wisconsin influenza virus [55]. Each subject includes 16 time point gene expression samples (Baseline, 0, 5, 12, 21, 29, 36, 45, 53, 60, 69, 77, 84, 93, 101, and 108 h). The gene expression profiles are measured on whole peripheral blood at an interval of 8 h post-inoculation (hpi) through 108 hpi. We only analyze 14 subjects because the other 2 subjects lack data at some time points. The 14 subjects include 7 subjects with clinical symptoms of influenza infection and 7 subjects without any clinical symptom at all-time points.

The lung adenocarcinoma (LUAD) dataset includes 518 tumor samples and 58 tumor-adjacent samples. The tumor samples are divided into different stages based on clinical (stage) information from TCGA. Based on clinical information, the samples are grouped into ten stages, i.e., stage I, IA, IB, II, IIA, IIB, IIIA, IIIB, and IV of lung cancer (Table 1).

**Table 1:** The number of tumor samples within each stage in the LUAD dataset from TCGA

Stage	I	IA	IB	II	IIA	IIB	IIIA	IIIB	IV	TA
Sample	5	134	147	1	50	71	73	11	26	58

The dataset GSE2565 (https://www.ncbi.nlm.nih.gov/geo/query/acc.cgi?acc=GSE2565) comprises expression profiles of the mouse with acute lung injury induced by phosgene inhalation. These lung tissues are collected from air- or phosgene-exposed mice at 0, 0.5, 1, 4, 8, 12, 24, 48, and 72 h after exposure (Table 2) [54]. RNA is extracted from the lung and used as starting material for the probing of oligonucleotide microarrays to determine changes in gene expression following phosgene exposure.

**Table 2:** The number of samples within each hour in the dataset GSE2565

Hour	0	0.5	1	12	24	4	48	72	8
Sample	6	12	12	12	12	12	14	12	12

### Algorithm to detect the pre-disease state based on SSP

Because it is hard to characterize the health degree of an individual using one time point expression data, Chen et al. developed a novel framework that indirectly depicts the deviation of an individual at time point ${\text{t}}$ from the health state based on the Pearson correlation changes between two groups of samples. Specifically, the first is the reference group composed of $n$ samples with a normal/healthy state, the other is the mixed group composed of the $n$ reference samples and one sample at a time point ${\text{t}}$. Given gene $g_{i}$ and its $k$ th neighbor gene $g_{ik}$ (total $M$ neighbor genes) in a PPI network, the local entropy of the gene $g_{i}$ at time point t is defined as

1 $$\it {\text{H}}^{n} (g_{i} ,t) = - \frac{1}{\log M}\sum\limits_{k = 1}^{M} {p_{k}^{n} (t)\log } p_{k}^{n} (t)$$

with

2 $$p_{k}^{n} (t) = \frac{{\left| {PCC^{n} (g_{i}^{n} (t),g_{ik}^{n} (t))} \right|}}{{\sum\limits_{j = 1}^{M} {\left| {PCC^{n} (g_{i}^{n} (t),g_{ij}^{n} (t))} \right|} }}$$

where $PCC^{n} (g_{i} (t),g_{ik} (t))$ represents the Pearson Correlation Coefficient between the gene $g_{i}$ and the $k$ th neighbor gene $g_{ik}$ based on $n$ reference samples. $g_{i}^{n} (t)$ and $g_{ik}^{n} (t)$ respectively denote the expressions of genes $g_{i}$ and $g_{ik}$ at time point ${\text{t}}$ based on $n$ reference samples. Then for a single sample at the time point ${\text{t}}$, SLE mixes it with the $n$ reference samples. The local entropy $\it {\text{H}}^{n + 1} (g_{i} ,t)$ of the gene $g_{i}$ at the time point ${\text{t}}$ of the mixed $n + 1$ samples is calculated in a similar way to that in Eq. (1) and Eq. (2), but is based on the mixed $n + 1$ samples instead of n reference samples.

They also measured the expression perturbation of gene $g_{i}$ at time point ${\text{t}}$ by the differential standard deviation

3 $$\Delta SD(g_{i} ,t) = \left| {SD^{n + 1} (g_{i} ,t) - SD^{n} (g_{i} ,t)} \right|$$

where $SD^{n + 1} (g_{i} ,t)$ and $SD^{n} (g_{i} ,t)$ are the standard deviations of the gene $g_{i}$ respectively based on the reference samples and the mixed samples.

Then, the absolute differential entropy of the gene $g_{i}$ at the time point $t$ between the local entropies $H^{n + 1} (g_{i} ,t)$ and $H^{n} (g_{i} ,t)$ is weighted by $\Delta SD(g_{i} ,t)$ as

4 $$\Delta H(g_{i} ,t) = \Delta SD(g_{i} ,t)\left| {H^{n + 1} (g_{i} ,t) - H^{n} (g_{i} ,t)} \right|$$

Finally, the abnormal score of an individual at the time $t$ is the summation of $\Delta H(g_{i} ,t)$ for all $Q$ genes as

5 $$\Delta H(t) = \frac{1}{Q}\sum {\Delta H(g_{i} ,t)}$$

As the development of disease results from the abnormal molecular perturbation through some important signaling pathways, Tarca et al. proposed a signaling pathway impact analysis (SPIA) method to determine the significant disease-related signaling pathways by normal and disease samples [45]. They defined the signal perturbation $PF(g_{i} )$ of a gene $g_{i}$ in a specific signaling pathway as

6 $$PF(g_{i} ) = FC(g_{i} ) + \sum\limits_{k} {\beta_{ik} \frac{{PF(g_{k} )}}{{N_{k} }}}$$

where gene $g_{k}$ is the direct upstream gene of gene $g_{i}$ in the specific signaling pathway. $PF(g_{k} )$ is the signal perturbation of the gene $g_{k}$. $N_{k}$ is the number of the direct downstream genes of gene $g_{k}$. $\beta_{ik}$ is the strength of the interaction between gene $g_{k}$ and $g_{i}$. $\beta_{ik}$ is 1 when the interaction between the two genes is activated. $\beta_{ik}$ is −1 when the interaction between the two genes is inhibited. $FC(g_{i} )$ is the fold change of the expression of the gene $g_{i}$ by normal and disease samples.

Signaling pathways in the KEGG database can transmit the extracellular molecular signals into cellcells to exert effects through specific biological functions. Complex diseases always associate with the abnormal biological functions that direct impacted by the most downstream genes. The accumulation of differential expressions and signal perturbations in signaling pathways is an invertible dynamic process before the biological functions become uncontrolled. The upstream genes in signaling may be very differential expression in the pre-disease state. As demonstrated by SPIA, the amount of signal perturbations in signaling pathways are directly impact by the expression value of upstream genes. Therefore, following SPIA, we adapt the dynamic time-series expression data to static signaling pathwaysin the same wayinstead of the correlations calculated in SLE.

7 $$PF(g_{i} ,t) = FC(g_{i} ,t) + \sum\limits_{k} {\beta_{ik} \frac{{PF(g_{k} ,t)}}{{N_{k} }}}$$

where gene $g_{k}$ is the direct upstream gene of gene $g_{i}$ in the specific signaling pathway. $PF(g_{i} ,t)$ is the signal perturbation of the gene $g_{k}$ at a time point t. $N_{k}$ is the number of the direct downstream genes of gene $g_{k}$ in the specific signaling pathway. $\beta_{ik}$ is the strength of the interaction between gene $g_{i}$ and $g_{k}$. $\beta_{ik}$ is 1 when the interaction between the two genes is activated. $\beta_{ik}$ is −1 when the interaction between the two genes is inhibited. $\it {\text{FC}}(g_{i} ,t)$ is the fold change of the gene $g_{i}$ at a time point ${\text{t}}$.

8 $$FC(g_{i} ,t) = \log_{2} \frac{{g_{i} (t)}}{{\overline{E}}}$$

$g_{i} (t)$ is the expression of the gene $g_{i}$ at the time point ${\text{t}}$. $\overline{E}$ is the average expression of all $n$ reference samples.

Then, the weighted signal perturbation (SSP score) for all genes in all signaling pathways at the time point ${\text{t}}$ is calculated, i.e.

9 $$\Delta {{PF}}(t) = \sum {\left| {PF(g_{i} ,t)} \right|} \cdot \Delta SD(g_{i} ,t)$$

**Fig. 7:**
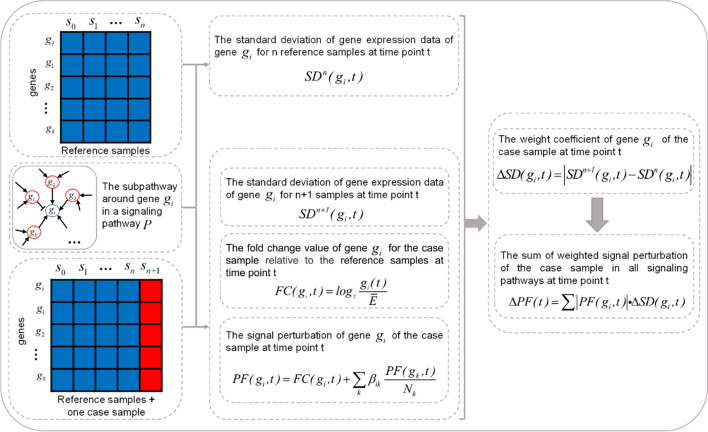
The schematic illustration of the SSP method

If the SSP score has a sharp increase from the previous time point to a time point ${\text{t}}$, the time point ${\text{t}}$ will be considered as an early-warning signal for the disease. Figure 7 shows the schematic diagram of the proposed SSP method. The main difference between SSP and SLE based method is that SSP directly describes the deviation of an individual at a time point from a normal state by adapting the signal perturbation transmitted in static signaling pathways instead of the indirect way in SLE.

## Data Availability

SSP is available and open source at https://github.com/ZhenshenBao/SSP. The datasets used and/or analyzed during the current study are available and are listed in the manuscript.

## Abbreviations

SLE: Single-sample landscape entropy
PPI: Protein- Protein Interaction
SSP: Single-sample signal perturbation
DNB: Dynamic network biomarker
SPIA: Signaling pathway impact analysis
LUAD: Lung adenocarcinoma
BALF: Bronchi alveolar lavage fluid
TA: Tumor adjacent
